# Supplementary material for: Understanding the Role of External Facilitation to Drive Quality Improvement for Stroke Care in Hospitals
Source: Healthcare (Basel). 2021 Aug 25;9(9):1095. doi: 10.3390/healthcare9091095 (PMC8471416; doi:10.3390/healthcare9091095)
Supplement: Supplementary file 1 [file healthcare-09-01095-s001.zip › healthcare-1326889-supplementary.pdf]

Online Supplement

Understanding the Role of External Facilitation to Drive Quality Improvement for Stroke Care in Hospitals

Tharshanah Thayabaranathan [1,*], Nadine E. Andrew [1,2], Rohan Grimley [1,3,4], Enna Stroil-Salama [5], Brenda Grabsch [6], Kelvin Hill [7], Greg Cadigan [3], Tara Purvis [1], Sandy Middleton [8], Monique F. Kilkenny [1,6], Dominique A. Cadilhac [1,6] and on behalf of the Stroke123 Investigators and AuSCR Consortium [†]

[1] School of Clinical Sciences at Monash Health, Monash University, Clayton, VIC 3168, Australia; nadine.andrew@monash.edu (N.E.A.); r.grimley@griffith.edu.au (R.G.); tara.purvis@monash.edu (T.P.); Monique.Kilkenny@monash.edu (M.F.K.); dominique.cadilhac@monash.edu (D.A.C.)

[2] Peninsula Clinical School, Central Clinical School, Monash University, Frankston, VIC 3199, Australia

[3] Queensland State-Wide Stroke Clinical Network, Brisbane, QLD 4000, Australia; Greg.Cadigan@health.qld.gov.au

[4] Sunshine Coast Clinical School, Griffith University, Birtinya, QLD 4575, Australia

[5] Metro South Research, Metro South Health, Brisbane, QLD 4102, Australia; Enna.Stroil-Salama@health.qld.gov.au

[6] Stroke Division, The Florey Institute of Neuroscience and Mental Health, Heidelberg, VIC 3052, Australia; brenda.grabsch@gmail.com

[7] Stroke Foundation, Melbourne, VIC 3000, Australia; KHill@strokefoundation.org.au

[8] Nursing Research Institute, St Vincent's Health Network Sydney, St Vincent's Hospital Melbourne, Australia and Australian Catholic University, Sydney, NSW 2010, Australia; Sandy.Middleton@acu.edu.au

* Correspondence: tharshanah.thayabaranathan@monash.edu; Tel.: +61-3-8572-2646; Fax: +61-3-9902-4245

† The members of the collaborative group are listed in the Supplementary Material.

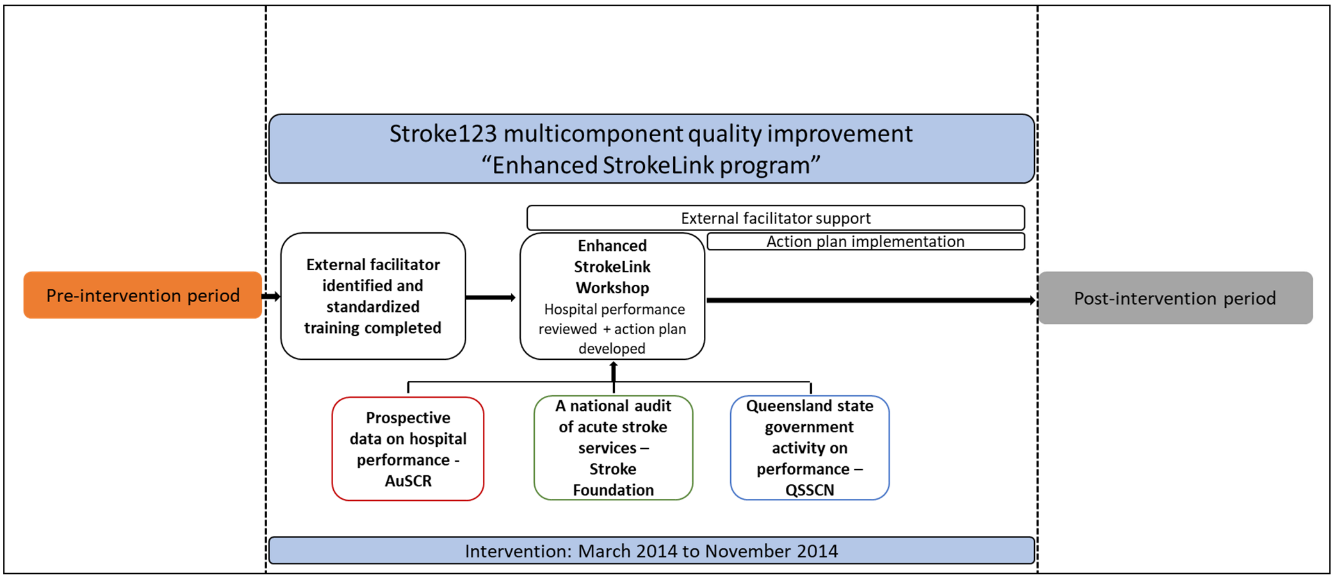

Figure S1. Overview of the Enhanced StrokeLink Program.
AuSCR: Australian Stroke Clinical Registry; QSSCN: Queensland State-wide Stroke Clinical Network; Pre-intervention: control with baseline audit and feedback via StrokeLink (January 2010 to June 2012) and addition of financial incentives, July 2012 to March 2014); Intervention: financial incentives and addition of the Enhanced StrokeLink program, March 2014 to November 2014; and Post-intervention period, November 2014 to December 2015), as outlined in the Figure.

Table S1. Example Enhanced StrokeLink Action Plan

**Team/Hospital Name:**	**Date:**
**Team Leader:**	
**Situation Statement:** Why initiate this effort? (key messages from guidelines and current activity based on audit, etc.)	
**Improvement AIMS**: What are we trying to accomplish? It is important to have clear goals	
**Improvement strategies/objectives:** What specific strategies have been chosen to bring about	
**Evaluation Measures:** How will we know a change is an improvement? Be specific about data source/measures and at what stages in the overall timeframes these will be collected and reported to keep team accountable and program on track.	
**Strategic Alignment (i.e. link to hospital plans/strategic directions):** Link current programs or specific activities to department, hospital and/or area administration plans. This will be important for administration support.	
**Consumer Alignment:** Who does this program relate to and how is the activity linked in with their needs? (E.g. Use local consumer data – surveys, focus groups, etc. or other data – NSF consumer consultation report 'walk in our shoes' or guidelines ratings)	
**Business Case:** Where possible, quantify expected increases or decreases in	

resources/expenses/reimbursement/cost avoidance.
**Team Members (and area of expertise):** Note also other key stakeholders who may not actually be on the core team but who will need to be involved.
**1.**
**2.**
**3.**
**4.**
**5.etc.**
**Implementation Commitments:** Note frequency and length of team meetings, resource implications (e.g. is there any training or support for the team?) and overall timeframes for project.
**Communication Plan:** Who, How, and When
**Signatures:**
Sponsor/manager: Date:
Team Leader/Champion: Date:

Supplemental acknowledgements

Co-investigators and other contributors to the Stroke123 project or Australian Stroke Clinical Registry (AuSCR) not listed as authors. Note only data obtained from 2010 – 2015 from AuSCR were used in this current paper, and from Queensland hospitals only.

Chief investigators: Prof **Craig Anderson** (University of New South Wales), Prof **Geoffrey Donnan** (University of Melbourne), Prof **Christopher Levi** (University of Necastle), Prof **Amanda Thrift** (Monash University), Prof **Natasha Lannin** (Monash University), Prof **Steven Faux** (University of New South Wales and St Vincent's Health), A/Prof **Vijaya Sundararajan** (University of Melbourne).

Associate investigators: Ms **Sonia Denisenko** (Victorian government); Professor **Helen Dewey** (Victorian government until 2014); Dr **Erin Godecke** (Western Australia Department of Health clinical representative); Ms **Judy Katzenellenbogen** (Department of Health data-linkage representative); Dr **Erin Lalor** (previously of the National Stroke Foundation); Dr **Andrew Lee** (Department of Health South Australia clinical representative); Mr **Mark Longworth** (New South Wales Agency for Clinical Innovation – Statewide Stroke Services Clinical Network up until 2015).

Others:
Queensland Statewide Stroke Clinical Network: Ms **Cindy Dilworth** (until 2013); Sarah Kuhle. **Stroke Foundation**: **Chris Price** (until December 2012); **Matthew Page** (Queensland StrokeLink Project Officer until 2013); **Helen Branagan** (Queensland StrokeLink Project Officer 2014- 2015), Ms **Jen Egan** (previously the Queensland National Stroke Foundation State Manager until 2014); Ms **Libby Dunstan** (previously the Queensland National Stroke Foundation State Manager until 2017), **Elizabeth Ritchie** (Queensland Quality Improvement and Evaluation sub-committee).

Monash University project staff: **Megan Reyneke and Joosup Kim**.

The following people are acknowledged for their contribution to collecting hospital data on the patients registered in AuSCR or their participation on various governance committees during the Stroke123 project.

Australian Stroke Clinical Registry Steering and Management Committees

Steering Committee representatives
Julie Bernhardt PhD (The Florey Institute of Neuroscience and Mental Health VIC); **Paul Bew** MPhty (The Prince Charles Hospital QLD); **Christopher Bladin** MD, MBBS, FRACP (Box Hill Hospital VIC, site investigator); **Helen Castley** MBBS (Royal Hobart Hospital Tasmania, site investigator); **Andrew Evans** MBBS (Hons), FRACP (Westmead Hospital NSW); **Susan Hillier** PhD (University of South Australia, SA); **Erin Lalor** PhD (previously employed by the National Stroke Foundation); **Andrew Lee** MBBS FRACP (Flinders Medical Centre, South Australia); **Richard Lindley** PhD (The George Institute for Global Health NSW); **Mark Mackay** MBBS, FRACP (Royal Children's Hospital VIC, site investigator); **Sandra Martyn** (Health Statistics Centre Queensland Health QLD); **John McNeil** PhD (Monash University VIC); **Michael Pollack** MBBS, FAFRM (RACP), FACRM, FFPM (ANZCA), MMedSci (Clin Epi) (Hunter Stroke Service NSW); **Mark Simcocks** BSc (VIC, Consumer Representative); **Frances Simmonds** MSc(Med), (Australasian Rehabilitation Outcomes Centre NSW); **Andrew Wesseldine** MBBS, FRACP (St John of God Healthcare; Department of Health WA).

Management Committee representatives: Helen Dewey PhD (Austin Hospital VIC, Box Hill Hospital VIC, site investigator, Eastern Health Clinical School, Monash University VIC); **Peter Hand*** MBBS, MD, FRACP (Royal Melbourne Hospital VIC, site investigator); **Christopher Price** BSocW BSc (previously employed by the National Stroke Foundation).
*on both committees

Queensland Hospital site investigators (Australian Stroke Clinical Registry) and registry staff
Pradeep Bambery MD, FRCP(G), FRACP (Bundaberg Hospital QLD); **Sean Butler** FIMLS, BM Hons, MRCP(UK), FRACP (Prince Charles Hospital QLD); **Carolyn De Wytt** MRCP (UK), MB BCH DUBL, FRACP (Greenslopes Private Hospital QLD); **David Douglas** MBBS, M Admin, FRACGP, FAFRM (RACP) (Ipswich Hospital QLD); **Martin Dunlop** MBBS, FACRM (Cairns Base Hospital QLD); **Paula Easton** BPhty (Hons) (Mackay Hospital QLD); **Nisal Gange** MBBS, AMC CERT (Toowoomba Hospital QLD); **Richard Geraghty** MBBS, FRACP (Redcliffe Hospital QLD); **Graham Hall** MBBS, FRACP (Princess Alexandra Hospital QLD); **Karen Hines** BHIM (Caboolture Hospital QLD); **Francis Hishon** RN (Redland Hospital QLD); **Joel Iedema** MBBS, FRACP (Redland Hospital QLD); **Paul Laird** MBBS, FRACP (Rockhampton Hospital QLD); **Graham Mahaffey** RN (Hervey Bay Hospital QLD); **Suzana Milosevic** MD, FRACP, AMC CERT (Logan Hospital QLD);; **Michaela Plante** RN (Div 1) (Rockhampton Hospital QLD); **Juan Rois- Gnecco** Medico Cirujano Javeriana, FAFRM (Ipswich Hospital QLD); **Stephen Read** MBBS, PhD, FRACP (Royal Brisbane and Women's Hospital QLD); **Arman Sabet** MD, FRACP, BSc (Gold Coast Hospital and Robina Hospital QLD); **Noel Saines** MBBS, FRACP (The Wesley Hospital QLD); **Eva Salud** MD, AMC CERT (Gympie Hospital QLD); **Amanda Siller** MBBS, FRACP (Queen Elizabeth II Jubilee Hospital QLD); **Christopher Staples** MD (Mater Adults QLD); **Richard White** MD, FRCP, FRACP. (Townsville Hospital QLD); **Andrew Wong** MBBS, PhD (Royal Brisbane and Women's Hospital QLD); **Lillian Wong** MBBS FRACP (Logan Hospital QLD).

AuSCR staff: the Florey Institute of Neuroscience and Mental Health (AuSCR Data Custodian from 2012)
Robin Armstrong†, Leonid Churilov, Alison Dias, Kelly Drennan, Adele Gibbs, Jen Holland, Joosup Kim (Monash University), Charlotte Krenus, Francis Kung, Karen Moss, Kate Paice, Enna Salama†, Sam Shehata, Renee Stojanovic†, Steven Street†, Emma Tod, Kasey Wallis, Julia Watt.

†During the Stroke123 study worked in the role of Queensland project coordinator and AuSCR coordinator
